# Recurrent isolated pulmonic valve infective endocarditis in a patient on chronic haemodialysis due to reinfection: description of an index case and management considerations

**DOI:** 10.1093/ehjcr/ytaf138

**Published:** 2025-03-24

**Authors:** Sapana Yonghang, Mitesh Karn, Ana Maritza Marulanda Prado, Ronny Cohen

**Affiliations:** NYC Health and Hospitals/Woodhull, 760 Broadway, Brooklyn, NY 11206, USA; Department of Medicine, Gandaki Medical College Teaching Hospital, Pokhara 33700, Nepal; NYC Health and Hospitals/Woodhull, 760 Broadway, Brooklyn, NY 11206, USA; NYC Health and Hospitals/Woodhull, 760 Broadway, Brooklyn, NY 11206, USA

**Keywords:** Pulmonic valve, Infective endocarditis, Haemodialysis, Arterio-venous graft, Case report

## Abstract

**Background:**

Right-sided infective endocarditis is uncommon, and isolated pulmonic valve infective endocarditis (PVIE) is rare. Chronic haemodialysis is a risk factor for developing infective endocarditis. but recurrent PVIE in this population is not well described.

**Case summary:**

A 32-year-old male with end-stage renal disease on chronic haemodialysis and diagnosed with PVIE 6 months back due to *Staphylococcus epidermidis* without identified source of infection presented again with recurrent PVIE with methicillin sensitive *S. aureus*, new pulmonic septic emboli, and peri-graft fluid collection on imaging, which was explanted with negative follow-up culture.

**Discussion:**

Through this index case, we describe the unique pathology of recurrent isolated PVIE and explore its diagnostic and management considerations.

**Learning points**

- We describe an index case of recurrent native pulmonic valve infective endocarditis (PVIE) due to reinfection from the same source.
- The diagnosis and management of PVIE in patients on haemodialysis can be challenging, especially in those with unidentified source of infection. Arterio-venous graft should always be thought of as the source in such cases.

## Introduction

Infective endocarditis (IE) is a major global public health problem and causes significant morbidity and mortality. The majority of cases of IE involves the left heart. Right-sided IE (RSIE) is relatively uncommon and accounts for <10% of all IE cases. Among people with RSIE, isolated pulmonic valve IE (PVIE) is an extremely rare occurrence and affects only 1.5–2% of these cases.^1^

Chronic haemodialysis (HD) is a long-known and well-established risk factor for developing IE. With a global increase in the end-stage renal disease (ESRD) burden, the number of patients requiring HD is increasing. Infective endocarditis is a serious and potentially lethal infection in patients undergoing HD, with the estimated overall prevalence reportedly being 2.9% in this group.^2^ In patients undergoing HD, the incidence of RSIE especially PVIE remains elusive.

Traditionally, recurrent episodes of IE have been divided into relapse and reinfection. Herein, we describe an index case of recurrent native PVIE in a patient undergoing HD, the recurrence attributed to reinfection by different bacteria to the same valve, which adds a layer to its rarity.

## Summary figure

**Table:** 

Date	Event
21 June 2023	History of admission for sepsis from possible infective endocarditis (IE). Echocardiography during that visit showed vegetation involving the pulmonic valve
23 June 2023	*Staphylococcus epidermidis* identified in culture report. Vancomycin, rifampin, and gentamicin initiated
26 June 2023	Blood culture negativity obtained
28 June 2023	Left against medical advice prior sensitivity report of the culture. Was given gentamicin for 2 weeks and vancomycin and rifampin for 6 weeks. The duration was counted from the date of negative blood culture
27 December 2023	Second admission for recurrent pulmonic valve IE with severe pulmonic regurgitation. Methicillin-sensitive *S. aureus* isolated from blood culture
3 January 2024	Left distal arteriovenous graft surveillance showed peri-graft fluid
9 January 2024	Explantation of the graft. Temporary haemodialysis access was placed. Subsequent blood cultures post-explant negative
16 January 2024	Discharged on cefazolin for 6 weeks

## Case presentation

A 32-year-old male with a history of hypertension, recurrent intravenous drug use, and ESRD on HD via left arterio-venous graft (AVG) three times a week presented to the emergency department with a 2-week history of subjective fever with chills, productive cough with whitish sputum, right-sided pleuritic chest pain, and rhinorrhoea. On presentation, he was febrile (temperature: 102.7°F/39.2°C) and tachycardic (heart rate 110 b.p.m.). Physical examination was notable for the left upper sternal diastolic murmur and healthy AVG site on the left upper extremity.

**Figure 1:**
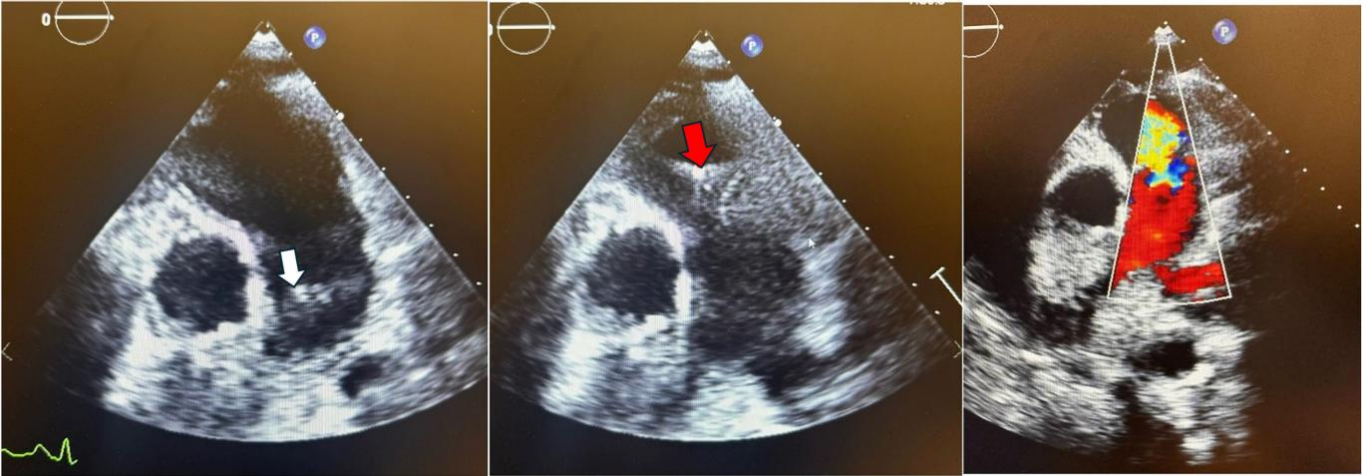
Parasternal short-axis echocardiogram with colour Doppler during the initial pulmonic valve infective endocarditis episode showing pulmonic valve vegetation during systole (white arrow), diastole (red arrow), and severe pulmonic regurgitation.

Six months back, the patient was diagnosed with native PVIE due to methicillin-sensitive *Staphylococcus epidermidis* (*Figure 1*). During that episode, he was initiated on vancomycin while awaiting sensitivity results due to concerns about heterotypic resistance of *S. epidermidis*. Though the AVG looked healthy, it was suspected as a possible source of IE. Due to the likelihood of infection at the prosthetic material–biological tissue interface, additional therapy with gentamicin and rifampin was also initiated. Regrettably, the patient left against medical advice before the sensitivity report was obtained, and surveillance to identify the infective source could not be carried out. The patient’s sensitivity test results subsequently indicated that the organism was sensitive to methicillin, but the change in antibiotics could not be communicated to the patient. He also missed outpatient follow-up appointments with vascular surgery for the evaluation of the graft. During the current visit, the HD centre was called to confirm the completion of antibiotic treatment during the initial episode, which consisted of a 2-week course of gentamicin and a 6-week regimen of a combination of vancomycin and rifampin.

**Figure 2:**
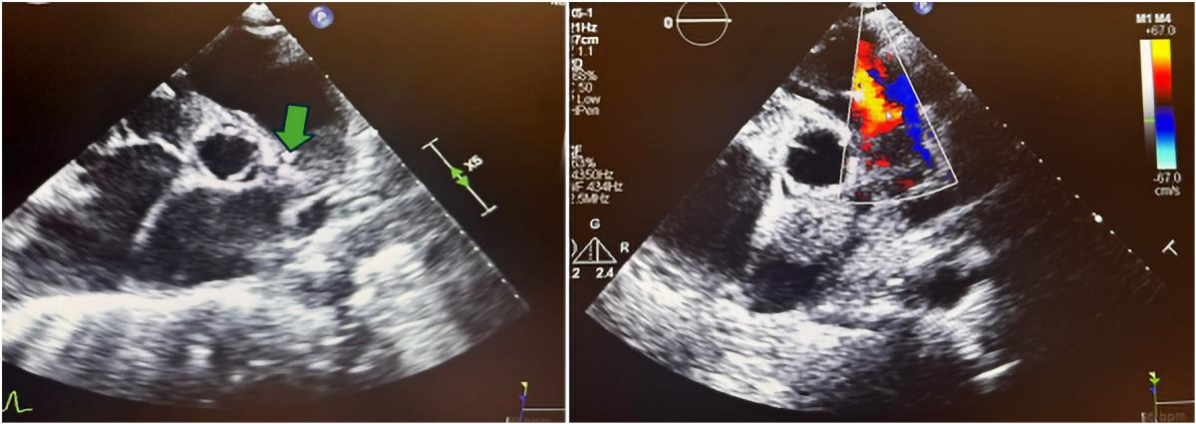
Parasternal short-axis echocardiogram with colour Doppler during the second pulmonic valve infective endocarditis episode showing vegetation on the arterial side of the pulmonic valve (arrow) and severe pulmonic regurgitation.

**Figure 3:**
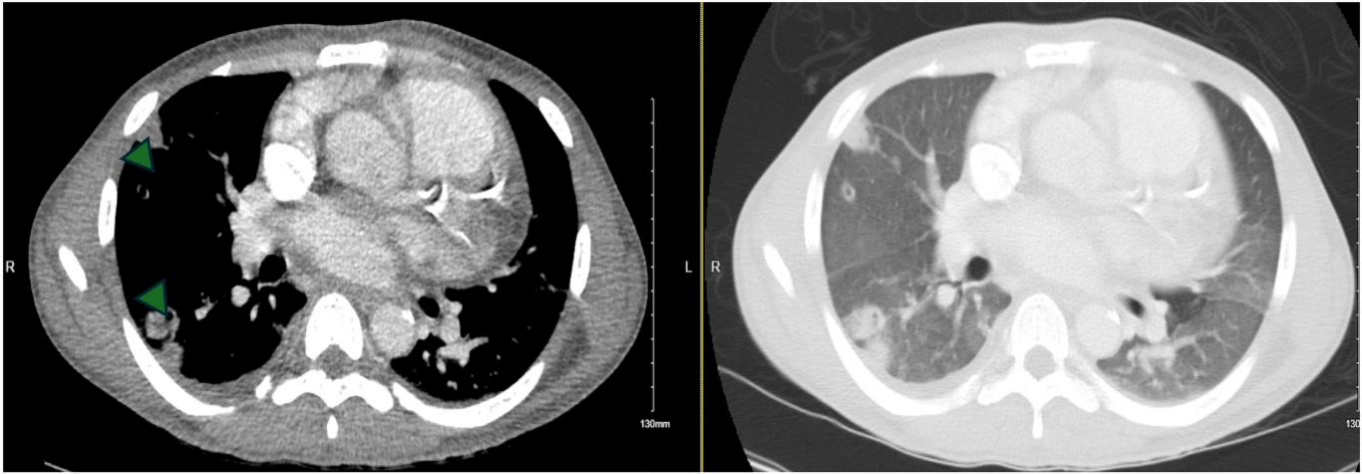
Computed tomography chest (axial slices) showing bilateral cavitary lung nodules (arrowheads) and ground glass opacities.

Blood work-up was significant for leucocytosis (WBC count 13 800/mm^3^), raised inflammatory markers: C-reactive protein 219 mg/L, procalcitonin 39.5 ng/dL, and lactate value of 2.2 mmol/L. Chest X-ray showed minimal left-sided pleural effusion. A provisional diagnosis of sepsis secondary to community-acquired pneumonia was made, and the patient was put up on vancomycin and piperacillin/tazobactam therapy. Provided prior history of PVIE, audible murmur on physical examination, and high-grade fever, IE was the main differential on our list. A transthoracic echocardiography was therefore ordered, which showed a 0.5 × 0.6 cm sessile, echogenic vegetation on the arterial side of pulmonic valve and severe pulmonic regurgitation (*Figure 2*). Chest computed tomography was also done for respiratory symptoms, which showed multiple bilateral cavitary nodules consistent with septic embolization and ground glass opacities suggesting pulmonary oedema (*Figure 3*).

**Figure 4:**
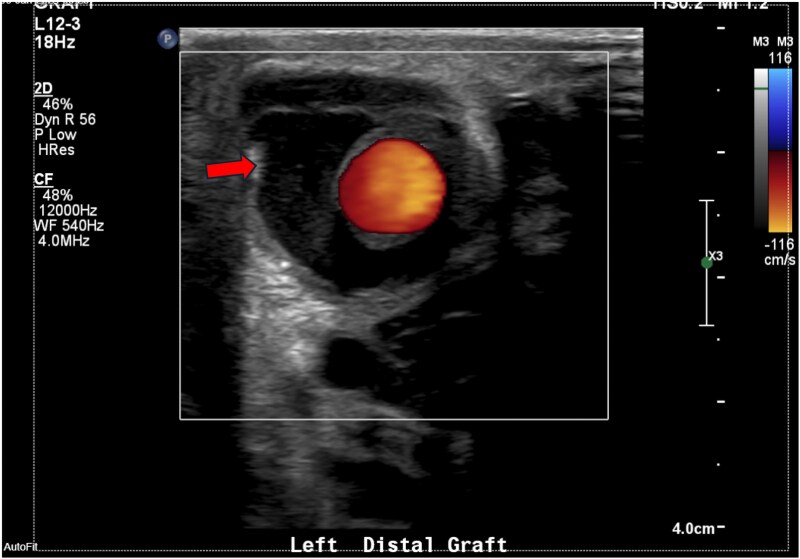
Ultrasonography of the graft site showing peri-graft fluid, ∼1.7 × 2.6 cm.

Blood cultures showed methicillin-sensitive *S. aureus*. Accordingly, the antibiotic regimen was changed to nafcillin 2 g every 4 h. Cardiothoracic surgery evaluation for PVIE-associated severe pulmonary regurgitation was carried out, but surgical intervention was not recommended. Despite antibiotic therapy, the bacteraemia was persistent. Graft surveillance was carried out, and graft site ultrasonography showed peri-graft fluid (*Figure 4*). A graft explant was therefore done following which blood cultures became negative. Right permcath was placed for temporary dialysis, and the patient was discharged on cefazolin for 6 weeks. Three weeks post-discharge, the patient had a follow-up with the infectious disease team and reported taking antibiotics as advised. A vascular surgery referral was made for creation of a new arterio-venous fistula.

## Discussion

Infective endocarditis in chronic HD (HD-IE) has recently been defined as a healthcare-associated infection with very high rates of mortality and relapses than in non HD-IE patients.^3^ Most cases of HD-IE occur in older adults and elderly females with the median reported age being around 60 years.^2,3^  *S. aureus* (including methicillin-resistant strains), enterococci, and coagulase-negative pathogens are more commonly implicated in HD-IE aetiology when compared with non HD-IE patients where viridans streptococci and *Streptococcus gallolyticus* are the common causative agents.^1,3^ Native pulmonic valve IE is uncommon even in the general population. In the HD cohort, synthesis of data from the International Collaboration on Endocarditis reports isolated pulmonic valve endocarditis as the least involved native valve endocarditis, accounting for only 0.5% of the cases.^3^

In our case, a young adult male was diagnosed as definite PVIE based on modified Duke’s major criteria: positive blood cultures and echocardiographic evidence of pulmonic valve vegetation with associated valvular regurgitation. In the first instance, coagulase-negative staphylococci—*S. epidermidis*—was isolated, but the source of infection could not be identified. During the recurrent PVIE episode, blood culture grew methicillin-sensitive *S. aureus.* Recurrent PVIE in HD patients has not been previously described. To the best of our knowledge, this is one of the rare instances of recurrent PVIE due to reinfection in this population.

The incidence of HD-IE is more than 50 times higher than in the general population. Several factors have been implicated in this increased risk, including repetitive access to the circulation, immunological susceptibility of patients with ESRD, and co-existing conditions such as valvular heart disease, diabetes mellitus, immunosuppressive therapy, and protein malnutrition.^4^ Type of HD access is also related to IE risk, the risk being highest with indwelling catheters and AVG. Recent evidence suggests that AV fistulas serve as the portal of entry in a high percentage of cases.^5^ The relapse rate in HD-IE is much higher, and HD is a permanent risk source for subsequent episodes of IE. In our case, the graft site showed no local signs of infection or growth on culture during both the infective episodes. Graft surveillance showed peri-graft fluid during reinfection, and the bacteraemia cleared only after graft explanation. Therefore, vascular access site must not be overlooked as a possible source of bacteraemia and IE, even if it does not appear infected. If the foci of infection are not found despite extensive search, the vascular access site must be presumed to be the infective focus.

Poor prognosis is expected in HD patients suffering from IE. About a third of HD-IE patients die during the first hospitalization, and the 1-year mortality rate is around 50%.^2^ Persistent bacteraemia, systemic embolization including septic pulmonary emboli, and heart failure are all associated with poor prognosis, and all these factors complicated the clinical picture in our case.^6^ Surgery for RSIE is commonly reserved for cases with very large vegetation (>20 mm), polyvalvular involvement, recurrent pulmonary emboli, and persistent sepsis.^3,6^ Since most of these criteria were not met and high peri-procedural risk in these patients, surgical intervention was deferred.

Pulmonary valve infective endocarditis is a rare entity associated with very high fatality rates.^7^ A high index of suspicion, prompt diagnosis, and proper management are paramount to prevent morbidity and mortality, especially if PVIE occurs in the setting of HD. Vascular access sites remain the commonest source of bacteraemia in this population, and every effort should be made to prevent access site infection through principles of asepsis. Any local sign of infection should warrant proper treatment, and surveillance should be carried out even if they appear healthy.

## Conclusion

Through this case, we describe a rare occurrence of recurrent native pulmonic valve IE possibly due to reinfection from the same source. Chronic HD is a well-known risk factor for IE, primarily due to access site infection and associated bacteraemia. A low threshold for suspicion of possible AVG infection despite no signs of infection at the catheter entry site can lead to early diagnosis and prompt management, thereby improving outcomes and preventing morbidity and mortality associated with PVIE in HD-IE population.

## Data Availability

The data underlying this article is available in the article itself.

## Lead author biography

I am a PGY3 Resident in NYC Health and Hospitals/Woodhull, Brooklyn and upcoming Chief Resident in NYC Health and Hospitals/Metropolitian. I love cardiology and aim to pursue fellowship in the same.

## Notes

**Consent:** The authors confirm that written consent for submission and publication of this case report, including images and associated text, has been obtained from the patient in line with COPE guidance.

**Funding:** None declared.
